# Effects of the commercial Chinese polyherbal preparation Zishen Yutai Pill on the pregnancy outcomes in women undergoing *in vitro* fertilization-embryo transfer: a systematic review and meta-analysis of randomized controlled trials

**DOI:** 10.3389/fphar.2026.1770841

**Published:** 2026-04-23

**Authors:** Haifeng Wu, Shuyi Ling, Zhisheng Zhong, Ruoxin Weng, Yuan Li, Wenbo Wu, Chongyang Ren, Mengying Bai, Liujuan Zhang, Yuehui Zheng

**Affiliations:** 1 The Fourth Clinical Medical College, Guangzhou University of Chinese Medicine, Shenzhen, China; 2 Shenzhen Traditional Chinese Medicine Hospital, Shenzhen, China

**Keywords:** *in vitro* fertilization-embryo transfer, meta-analysis, pregnancy outcomes, systematic review, Zishen Yutai Pill

## Abstract

**Aim of the Study:**

To systematically evaluate the effect of ZYP on pregnancy outcomes in women undergoing *in vitro* fertilization-embryo transfer (IVF-ET).

**Methods:**

Electronic databases (PubMed, Cochrane Library, Embase, Web of Science, CNKI, Wanfang, VIP, and CBM) were systematically searched from inception to 12 February 2025. The primary outcome measure was the clinical pregnancy rate. The secondary outcomes were live birth rate, biochemical pregnancy rate, miscarriage rate, implantation rate, endometrial thickness and number of oocytes retrieved. The ROB2 tool was used to assess the risk of bias, the data were pooled using Review Manager 5.3, and the pooled data were presented in terms of risk ratio (RR) or mean difference (MD) with a confidence interval of 95%. GRADE was used to evaluate the quality of the evidence. In addition, we performed sensitivity analysis, used Stata 17.0 for Egger’s test and constructed funnel plots to assess publication bias.

**Results:**

Eighteen RCTs (5,660 participants) were included. Compared to the control group, ZYP significantly improved the clinical pregnancy rate (RR = 1.22, 95% CI: 1.15–1.30, P < 0.00001), live birth rate (RR = 1.25, 95% CI: 1.15–1.36, P < 0.00001), biochemical pregnancy rate (RR = 1.13, 95% CI: 1.05–1.22, P = 0.002), and implantation rate (RR = 1.19, 95% CI: 1.11–1.27, P < 0.00001). Subgroup analysis suggested that the combination of biomedicine was more effective than ZYP alone. Additionally, ZYP was associated with a lower miscarriage rate (RR = 0.75, 95% CI: 0.63–0.88, P = 0.0005). ZYP also increased endometrial thickness on the day of transfer (MD = 1.06, 95% CI: 0.61–1.51, P < 0.00001) and the number of oocytes retrieved (MD = 0.77, 95% CI: 0.09–1.45, P = 0.03), though significant heterogeneity was observed for these two outcomes. The GRADE assessment indicated that the quality of evidence for all outcomes was very low. Regarding safety, only 5 of 18 studies reported adverse events. Among the 4 studies with quantifiable data (589 participants), all events were mild and self-limiting (e.g., nausea, constipation, rash), with incidence rates of 11.1% (27/244) in the ZYP group and 5.8% (20/345) in the control group. No serious adverse events were reported. Formal statistical comparison was not performed owing to the limited number of reporting studies and heterogeneous documentation methods.

**Conclusion:**

As an adjunctive treatment, ZYP appears to improve multiple pregnancy outcomes among women undergoing IVF. However, due to the limited certainty of evidence and the incomplete methodological reporting in the included studies, these findings do not support immediate changes in clinical practice and should be regarded as exploratory. Large-scale, double-blind RCTs with standardized ZYP protocols are needed to confirm these findings.

**Systematic Review Registration:**

https://www.crd.york.ac.uk/PROSPERO/view/CRD420251030089, identifier CRD420251030089.

## 1 Introduction

Infertility is a state of low fertility, referring to the failure to achieve clinical pregnancy after regular and non-contraceptive intercourse for at least 12 months (Chen et al., 2019). Its incidence varies according to countries, nationalities and regions, the global average is 8%–12%, but the average incidence in China is 25% and the incidence has been increasing year by year (Vander Borght and Wyns, 2018; Zhou et al., 2018). The higher incidence in China may relate to socioeconomic factors, childbearing and lifestyle (Zhou et al., 2018). This imposes substantial social and economic burdens, as infertility is associated with marital stress, healthcare costs, and reduced quality of life. The WHO has classified infertility as a public health priority in its 2023 Global Health Report (Macaluso et al., 2010). Assisted reproductive technology represented by IVF-ET is an important treatment for infertility, however, in most parts of the world, the live birth rate of IVF peaked in 2001–2002 and then gradually declined (Gleicher et al., 2019). Meanwhile the high proportion of transplant failure, adverse reactions and complications still cannot be ignored. Its overall clinical pregnancy rate in China was only 30.0%, and the live birth rate was 28.8%, similar to the data reported in the United States (27.3% and 22.2%, respectively) and Europe (29.3% and 22.3%, respectively) (Qiao et al., 2021). In other words, significant potential for improvement exists in the clinical pregnancy rate and live birth rate of IVF-ET. Addressing these limitations is critical to improving ART outcomes and patient wellbeing.

A study shows that in Ireland, 46% of infertile patients receive traditional Chinese medicine (TCM) treatment while undergoing IVF-assisted pregnancy to increase the success rate of pregnancy (Shannon et al., 2010). In TCM theory, the kidney governs reproduction, the occurrence of infertility is closely related to the kidneys, so the treatment mostly focuses on tonifying the kidneys (Zhao et al., 2024). The renowned commercial Chinese polyherbal preparation ZYP was formulated by late Professor Luo Yuankai, one of the first TCM professors in the People’s Republic of China, who was honored with the title “Grand Master of Gynecology in Lingnan”. ZYP is a commercial Chinese polyherbal preparation (CCPP) formulated as a honeyed pill and described in a pharmacopeial monograph with a fixed composition. In this review, the intervention is defined as oral administration of ZYP (alone or combined with biomedicine as reported), and the full clinical drug sources are presented in the Materials and Methods following best-practice reporting guidance. In TCM theory, ZYP aims to “fortify the spleen and nourish the kidneys”, which is thought to correspond to regulating the hypothalamic-pituitary-ovarian (HPO) axis. Previous studies have shown that ZYP can enhance endometrial receptivity and improve ovarian reserve and restore fertility by regulating the HOXA10 and AKT pathways (Chen et al., 2025; Dang et al., 2024; Gao et al., 2015). It is believed to possess clinical efficacy in the treatment of infertility and preventing pregnancy loss. At the same time, it has the advantages of convenience, standardization, painlessness and easy promotion. In recent years, with the development of assisted reproductive technology and the increasing incidence of infertility, ZYP has gradually been applied in the reproductive field. More and more researchers have observed that ZYP plays a positive role in the pregnancy outcome of IVF-ET (Chen et al., 2022b). But there is currently no systematic analysis summary of ZYP as an adjuvant treatment for IVF-ET. Therefore, in this study, a new systematic review and meta-analysis were conducted on the RCTs of ZYP assisting in the pregnancy outcome of women undergoing IVF-ET to evaluate its impact on the pregnancy outcomes of IVF-ET, with the aim of providing evidence-based medical support.

## 2 Methods

This systematic review and meta-analysis followed the PRISMA statement (Page et al., 2021) and was registered on the PROSPERO platform (registration number: CRD420251030089, https://www.crd.york.ac.uk/PROSPERO/view/CRD420251030089).

We characterised the investigated preparation using the ConPhyMP best-practice framework. ZYP is classified as a commercial Chinese polyherbal preparation formulated as honeyed pills with a fixed pharmacopeial composition defined in the Pharmacopoeia of the People’s Republic of China, 2020 Edition. It is produced exclusively by Guangzhou Baiyunshan Zhongyi Pharmaceutical Co., Ltd., under National Drug Approval No. Z44020008, ensuring that the product used across all included trials is pharmaceutically identical. Intervention details were extracted from each included trial report, covering dose, regimen, co-interventions, placebo or blinding status, and adverse-event reporting. Most trials, however, did not report specific batch numbers, and this is noted as a reporting limitation.

The complete composition of ZYP is presented in Table 1, which lists all 15 constituent drugs of both botanical and animal origin with validated taxonomy. Among them, the classification verification of plant drugs uses the 2020 Edition of the Pharmacopoeia of the People’s Republic of China (https://ydz.chp.org.cn/#/main) and Medicinal Plant Names Services (http://mpns.kew.org/mpns-portal/), while that of animal drugs uses Global Biodiversity Information Facility (https://www.gbif.org/) for classification verification.

**TABLE 1:** Chinese medicine taxonomic verification.

Kinds	No.	Traditional name (Pinyin)	Accepted scientific name & authority [family]	Plant/Animal part used	Role in traditional medicine (TCM)
Botanical drugs	1	Shu Di Huang	*Rehmannia glutinosa (Gaertn.) DC.* [Orobanchaceae]	Prepared root	Nourishes Yin and blood and enrich essence and marrow
	2	Sha Ren	*Wurfbainia longiligularis (T.L.Wu) Škorničk. and A.D.Poulsen* [Zingiberaceae]	Fruit	Regulate qi, eliminate dampness and stabilize the fetus
	3	Tu Si Zi	*Cuscuta chinensis Lam.* [Convolvulaceae]	Seed	Tonifies the liver and kidneys, consolidates essence and stabilizes the fetus
	4	Ren Shen	*Panax ginseng C.A.Mey.* [Araliaceae]	Root and rhizome	Tonifies original Qi
	5	Sang Ji Sheng	*Taxillus chinensis (DC.)* Danser [Loranthaceae]	Aerial parts	Nourishes liver and kidney, and stabilizes the fetus
	6	He Shou Wu	*Pleuropterus multiflorus (Thunb.) Turcz. ex Nakai* [Polygonaceae]	Root	Tonifies the liver and kidneys, and nourishes blood and essence
	7	Ai Ye	*Artemisia argyi H.Lév. and Vaniot* [Asteraceae]	Leaf	Warms meridians and stops bleeding
	8	Ba Ji Tian	*Gynochthodes officinalis (F.C.How) Razafim. and B.Bremer* [Rubiaceae]	Root	Tonifies kidney Yang
	9	Bai Zhu	*Atractylodes macrocephala Koidz.* [Asteraceae]	Rhizome	Strengthens the spleen, benefit qi and stabilize the fetus
	10	Dang Shen	*Codonopsis pilosula (Franch.) Nannf.* [Campanulaceae]	Root	Strengthens the spleen and lungs, nourish the blood and generate body fluids
	11	Gou Qi Zi	*Lycium barbarum L.* [Solanaceae]	Fruit	Nourishes liver and kidney, and enhance essence
	12	Xu Duan	*Dipsacus asper Wall. ex DC.* [Caprifoliaceae]	Root	Tonifies liver and kidney
	13	Du Zhong	*Eucommia ulmoides Oliv.* [Eucommiaceae]	Bark	Tonifies the liver and kidneys and stabilizes the fetus
Animal-derived products	14	E Jiao	*Equus asinus Linnaeus* [Equidae]	Donkey-hide gelatin	Nourishes blood and Yin
	15	Lu Jiao Shuang	*Cervus nippon Temminck/Cervus elaphus Linnaeus* [Cervidae]	Degelatinized deer antler	Warms kidney Yang

### 2.1 Inclusion criteria

**P** (population): There were no restrictions on participants and all indications for IVF were included, including but not limited to PCOS (polycystic ovary syndrome), DOR (diminished ovarian reserve), tubal infertility, etc. There were no restrictions on age, the duration of infertility, or a history of previous implantation failure. It is unlimited for fresh and frozen embryos.

**I** (intervention): The experimental group was treated with ZYP alone or combined with conventional biomedicine on the basis of IVF-ET, and the taking time of ZYP was unrestricted.

**C** (comparators): The control group received either placebo, routine IVF-ET protocols without ZYP, or the same conventional biomedicine co-interventions as the experimental group but without ZYP.

**O** (outcomes): The primary outcome was the clinical pregnancy rate, and the secondary outcomes were the live birth rate, biochemical pregnancy rate, miscarriage rate, embryo implantation rate, endometrial thickness on the day of ET and the number of oocytes retrieved.

**S** (study design): Only randomized controlled trials of ZYP with the outcome of IVF-ET clinical pregnancy rate published in Chinese or English in journals were included in this review.

### 2.2 Exclusion criteria

The following were not included in this review: a) The results did not include clinical pregnancy rates. b) Conference papers, reviews, animal experiments, retrospective studies, case-control studies, etc., c) RCTs with no more than 20 participants in the experimental group.

### 2.3 Search strategy

We searched electronic databases including Embase, PubMed, Cochrane Library, Web of Science, CBM, CNKI, Wanfang, and VIP for RCTs from inception to 12 February 2025. The search terms included “(‘Zishen Yutai’ OR ‘ZYP’) AND (‘*in vitro* fertilization’ OR ‘IVF-ET’ OR ‘embryo transfer’)”. If the data of the research registration platform were not accessible, displaying in grey, we did not include this part into our study. Detailed search strategies are in the Supplementary Material 1.

### 2.4 Study screening

Two researchers (RW and MB) conducted literature searches and imported EndNote X9 according to the search strategy search and further manual procedures to remove duplicates, respectively. After removing duplicates, articles were filtered by title and abstract. Based on the inclusion criteria, two researchers performed full-text reading and finally identified eligible articles. If there are any differences, they are solved by the third researcher (ZZ).

### 2.5 Data extraction

Two researchers (WW and CR) independently extracted data according to the pre-designed table, and cross-checked the data after the extraction. If there was any disagreement, the third researcher (YZ) would solve the problem. The extracted data include: first author, publication year, sample size, intervention plan, outcome indicators, etc.

Among them, the research outcome indicators include: a) Clinical pregnancy rate. Clinical pregnancy refers to intrauterine gestational sacs with fetal heart detected by transvaginal ultrasound scan 4–6 weeks after embryo transfer. b) Live birth is defined as giving birth to a live baby after 28 weeks of gestation. c) Biochemical pregnancy refers to “a pregnancy diagnosed only by the detection of beta hCG in serum or urine (Zegers-Hochschild et al., 2017). d) Miscarriage: pregnant abortion all 28 weeks ago; e) Embryo implantation rate. Embryo implantation rate was defined as the number of implanted embryos divided by the total number of embryos transferred. (number of gestational sacs/Number of transferred embryos) × 100%. f) Endometrial thickness g) Number of oocytes retrieved.

### 2.6 Risk of bias assessment

The quality of the published literature was evaluated independently by two researchers (HW and SL) using the Cochrane Risk Bias assessment tool (Sterne et al., 2019). The study was evaluated in five areas: randomization process, deviation from intended intervention, missing outcome data, measurement of outcome, and selection of reported results. Each domain was evaluated as having a high risk of bias, some concerns or low risk of bias. Any disagreements were resolved by consensus or consultation with a third researcher (YZ).

### 2.7 Data summary and analysis

We used Review Manager 5.3 to summarize and analyze the data. Outcome measures were expressed as risk ratio (RR) for binary data, mean differences (MD) for continuous data, and with 95% confidence intervals (CI). The results were presented as forest plots. Subgroup analysis was conducted on the clinical pregnancy rate based on whether biomedicine was combined to evaluate potential differences in effect sizes between the two treatment approaches. Statistical heterogeneity among included studies was evaluated using the I2 statistic and the p value of the chi-square (χ^2^) test. If both P ≥ 0.05 and I^2^ ≤ 50% were met, heterogeneity was considered low, and a fixed-effect model was adopted; otherwise, a random-effect model was used. Furthermore, we performed sensitivity analysis using the leave-one-out method. When the number of trials reached 10 or more, a funnel plot and Egger’s test performed by Stata 17.0 was used to assess publication bias.

### 2.8 Quality of evidence

We adopted GRADE (Andrews et al., 2013) to assess the confidence in the available evidence for each outcome. Two researchers (HW and SL) independently evaluated the overall evidence quality by assessing each outcome’s risk of bias, inconsistencies in included trials, and indirectness and imprecision of the combined effect estimation. The evidence quality was classified as high, medium, low or very low. All areas of disagreement were resolved through consultation by the third researcher (ZZ).

## 3 Results

### 3.1 Study selection and characteristics

**FIGURE 1:**
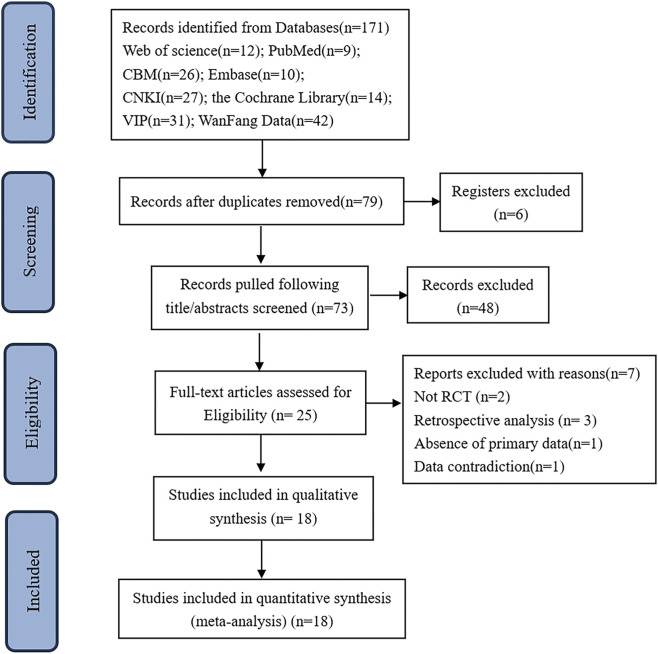
The screening flowchart.

A total of 171 studies were retrieved. After eliminating duplicates, 79 remained; 6 were removed for registration reasons, leaving 73 studies. Following screening of titles and abstracts, 48 studies were excluded, resulting in 25 studies for full-text review. Seven studies were further excluded: 3 were retrospective studies (Zhang et al., 2024; Zhang et al., 2023; Zhu, 2023), 2 non-RCTs (Li et al., 2019; Yao et al., 2020a), 1 lacked necessary outcome indicators (Yao et al., 2020b), and 1 had inconsistent data (Bo et al., 2018). Finally, 18 RCTs were included. The literature screening flow chart is shown in Figure 1.

Eighteen RCTs were conducted in China, published in Chinese or English, involving a total of 5,660 patients with IVF-ET, with a minimum sample size of 60 (Wang et al., 2024) and a maximum sample size of 2,265 (Chen et al., 2022a). Two studies were multicenter studies (Chen et al., 2022a; Chen et al., 2024). The duration of oral administration of ZYP varies from 3 weeks to 3 months. Fourteenstudies evaluated ZYP monotherapy (Cao et al., 2021; Chen et al., 2022a; Chen et al., 2024; Gao, 2016; Guo et al., 2024; Jinghua et al., 2024; Li et al., 2023; Lin and Wang, 2024; Wang et al., 2024; Wang et al., 2015; Yang and Yao, 2017; Yao et al., 2018; Zhou, 2015; Zhu et al., 2002), and 4 investigated ZYP combined with biomedicine. Specifically, one study combined ZYP with aspirin (Wu et al., 2016), two combined ZYP with atosiban (Yang and Li, 2023; Zhang and Liu, 2019), and one combined ZYP with phloroglucinol (Liu and Liu, 2020). Specific characteristics of the included studies are shown in Table 2.

**TABLE 2:** Characteristics of included studies.

Included studies	Sample (I/C)	Mean age/years (I/C)	Characteristics of the participants	Intervention	Comparison	Outcomes	Adverse events
Cao et al. (2021)	94 (47/47)	37.10 ± 8.09/36.23 ± 7.92	Infertility duration of ≥3 years	ZYP	No adjuvant treatment	①④	Nausea vomiting, joint pain, diarrhea, rash
Chen et al. (2022a)	2,265 (1131/1134)	30.7 ± 4.2/30.7 ± 4.1	Fresh embryo transfer	ZYP	Placebo	①②③④⑤⑥⑦	NR
Chen et al. (2024)	880 (441/439)	31.58 ± 4.33/31.65 ± 4.45	Frozen embryo transfer	ZYP	Placebo	①②③④⑤⑥	NR
Gao (2016)	100 (50/50)	NR	Tubal obstructive infertility	ZYP	No adjuvant treatment	①④⑥⑦	NR
Guo et al. (2024)	100 (50/50)	31.52 ± 5.03/30.51 ± 4.98	Fresh embryo transfer	ZYP	Placebo	①④⑥⑦	NR
Li et al. (2023)	120 (60/60)	33.55 ± 3.99/33.87 ± 3.50	DOR	ZYP	No adjuvant treatment	①②③④⑤⑥⑦	NR
Lin and Wang (2024)	116 (58/58)	33.11 ± 1.12/32.12 ± 1.01	RIF	ZYP	No adjuvant treatment	①②④⑥	NR
Liu and Liu (2020)	160 (80/80)	32.17 ± 6.09/31.75 ± 6.01	RIF	ZYP + phloroglucinol	Phloroglucinol	①②③④⑥	NR
Wang et al. (2024)	60 (30/30)	29.30 ± 2.71/28.97 ± 2.99	PCOS of kidney deficiency type	ZYP	No adjuvant treatment	①⑥⑦	No adverse reactions
Wang et al. (2015)	100 (50/50)	NR	Frozen embryo transfer	ZYP	No adjuvant treatment	①⑥	NR
Wu et al. (2016)	580 (360/220)	31.20 ± 3.85/31.05 ± 3.32	IVF	ZYP + aspirin	Aspirin	①②④⑤⑥⑦	NR
Yang and Li (2023)	88 (44/44)	29.54 ± 3.58/29.85 ± 3.47	RIF	ZYP + atosiban	Atosiban	①②④⑥	NR
Yang and Yao (2017)	140 (70/70)	NR	Kidney qi deficiency type	ZYP	No adjuvant treatment	①④⑥⑦	NR
Yao et al. (2018)	72 (36/36)	31.35 ± 4.06/30.63 ± 3.75	A history of biochemical pregnancy	ZYP	No adjuvant treatment	①④⑤⑥⑦	NR
Zhang et al. (2024)	287 (90/197)	34.53 ± 4.51/34.40 ± 4.22	DOR patients undergoing frozen embryo transfer	ZYP	Placebo	①②③④⑤⑥⑦	No adverse reactions
Zhang and Liu (2019)	210 (105/105)	29.99 ± 3.87/29.95 ± 3.83	RIF	ZYP + atosiban	Atosiban	①②④⑤⑥	NR
Zhou (2015)	148 (77/71)	30.96 ± 3.7/31.62 ± 4.73	IVF	ZYP	Placebo	①⑥⑦	Dry heat, constipation, acne, dry mouth, internal heat
Zhu et al. (2002)	140 (70/70)	31.7 ± 5.9/31.3 ± 6.2	IVF/ICSI	ZYP	No adjuvant treatment	①⑤⑥⑦	Constipation

① Clinical pregnancy rate ② Live birth rate ③ Biochemical pregnancy rate ④ Miscarriage rate ⑤ Implantation rate ⑥ Endometrial thickness ⑦ Number of oocytes retrieved. DOR: diminished ovarian reserve, RIF: recurrent implantation failure, PCOS: polycystic ovary syndrome, IVF: *in vitro* fertilization, ICSI: intracytoplasmic sperm injection, ZYP: zishen yutai pill.

### 3.2 Risk of bias

**FIGURE 2:**
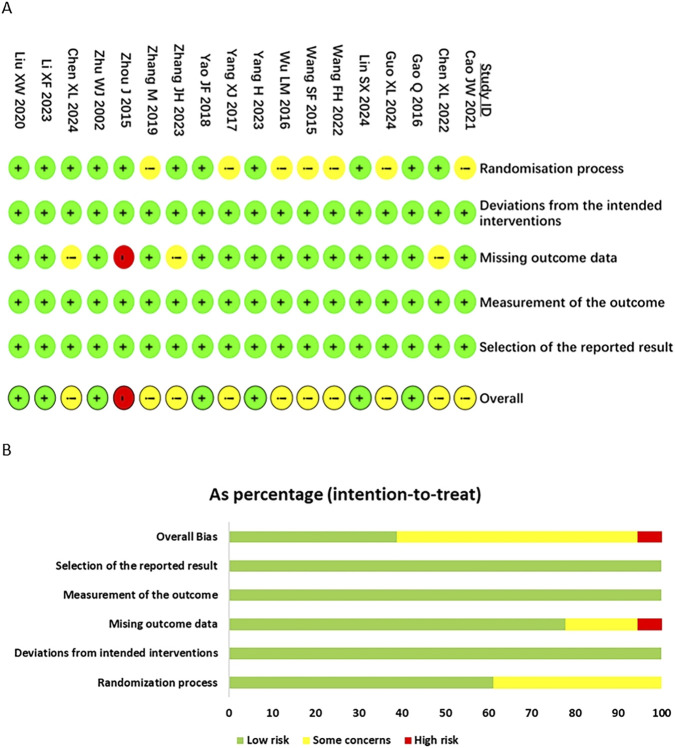
**(A)** Risk of bias item for included RCTs. **(B)** Risk of bias item presented as percentages across all included RCTs.

All RCTs mentioned “random assignment”, of which 7 studies (Chen et al., 2022a; Gao, 2016; Jinghua et al., 2024; Lin and Wang, 2024; Liu and Liu, 2020; Yao et al., 2018; Zhou, 2015) used random number table method, 2 studies (Chen et al., 2024; Yang and Li, 2023) used computer randomization method, 1 study (Li et al., 2023) used “permutation block randomization” method, and 1 study (Zhou, 2015) only described “random table” method. The remaining 7 studies (Cao et al., 2021; Guo et al., 2024; Wang et al., 2024; Wang et al., 2015; Wu et al., 2016; Zhang and Liu, 2019; Yang and Yao, 2017) only provided a brief description “randomized” and were rated as “some concerns”. 4 studies (Chen et al., 2022a; Chen et al., 2024; Jinghua et al., 2024; Zhou, 2015) implemented double-blind, 3 studies (Chen et al., 2022a; Chen et al., 2024; Jinghua et al., 2024) had allocation concealment, and none of the remaining studies mentioned double-blind and allocation concealment. Since this review assesses pregnancy outcomes, including clinical pregnancy rate, live birth rate, biochemical pregnancy rate, and miscarriage rate, the measurement methods of these results are objectively fixed and not easily affected by the subjective influence of the assessors. Therefore, allocation bias, measurement bias, and reporting bias were rated as low risk. Among the risks of dropout bias, 1 study (Zhou, 2015) was rated as high-risk due to a dropout rate of over 20% and no processing of missing data. 2 studies (Chen et al., 2022a; Chen et al., 2024) had a dropout rate of over 5% but were rated as some concerns using “intention-to-treat” (ITT) analysis, and 1 study (Jinghua et al., 2024) was rated as some concerns because the dropout rate in the experimental group (10%) was much higher than that in the control group (1.5%) and no ITT analysis was used. The evaluation of research quality is shown in Figure 2.

### 3.3 Outcomes

#### 3.3.1 Primary outcomes

**FIGURE 3:**
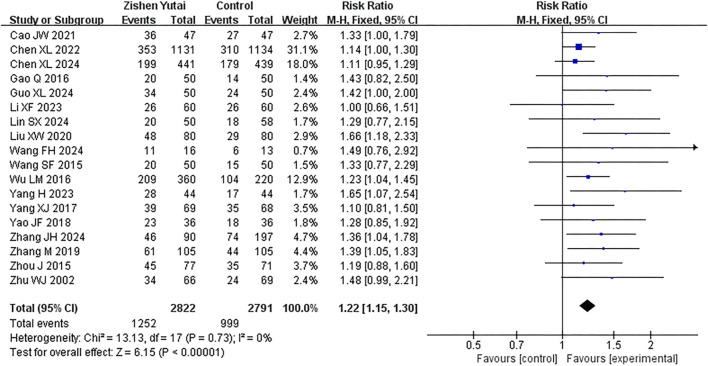
Forest plot of clinical pregnancy rate.

**FIGURE 4:**
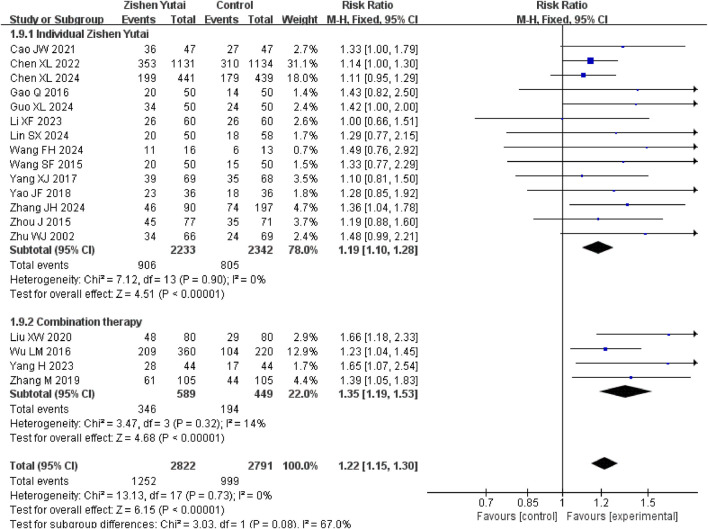
Forest plot of subgroup analysis on clinical pregnancy rate.

All the 18 RCTs reported the clinical pregnancy rate, and the statistical heterogeneity of all the studies was low (I2 = 0%, P = 0.73), so the fixed effect model was used. The results showed that ZYP provided significant overall benefits in improving the clinical pregnancy rate of IVF-ET patients (RR = 1.22,95% CI:1.15–1.3, P < 0.00001) (Figure 3). Subgroup analysis indicated that combining ZYP with biomedicine yields a greater therapeutic effect than using ZYP alone (Figure 4).

#### 3.3.2 Secondary outcomes

**FIGURE 5:**
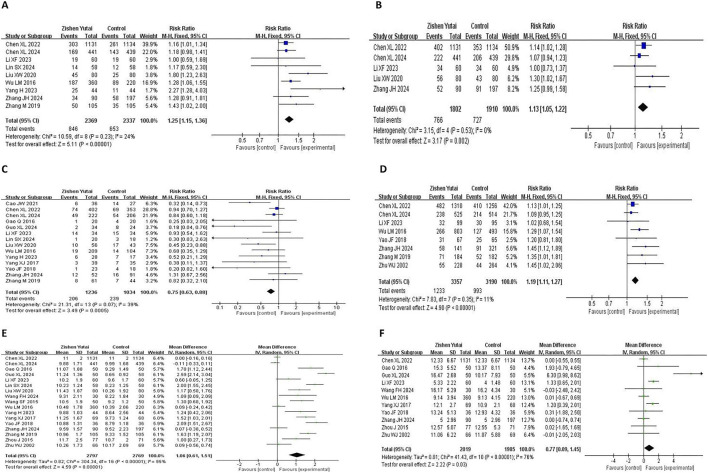
Forest plots of secondary outcomes. **(A)** Live birth rate; **(B)** Biochemical pregnancy rate; **(C)** Miscarriage rate; **(D)** Implantation rate; **(E)** Endometrial thickness on the day of embryo transfer; **(F)** Number of oocytes retrieved.

Nine (Chen et al., 2022a; Chen et al., 2024; Jinghua et al., 2024; Li et al., 2023; Lin and Wang, 2024; Liu and Liu, 2020; Wu et al., 2016; Yang and Li, 2023; Zhang and Liu, 2019) studies reported live birth rates, and compared with the control group, ZYP increased the live birth rate (RR = 1.25, 95%CI: 1.15–1.36, P < 0.00001) with low heterogeneity (I2 = 24%, P = 0.23) (Figure 5A). 5 studies (Chen et al., 2022a; Chen et al., 2024; Jinghua et al., 2024; Li et al., 2023; Liu and Liu, 2020) found that ZYP was superior to the control group in increasing the biochemical pregnancy rate (RR = 1.13,95% CI:1.05–1.22, P = 0.002), and the heterogeneity was also relatively low (I2 = 0%, P = 0.53) (Figure 5B). Fourteen studies (Cao et al., 2021; Chen et al., 2022a; Chen et al., 2024; Gao, 2016; Guo et al., 2024; Jinghua et al., 2024; Li et al., 2023; Lin and Wang, 2024; Liu and Liu, 2020; Wu et al., 2016; Yang and Li, 2023; Yang and Yao, 2017; Yao et al., 2018; Zhang and Liu, 2019) reported miscarriage rate, which showed an advantage in reducing miscarriage rates compared with the control group (RR = 0.75, 95% CI: 0.63–0.88, P = 0.0005), with low heterogeneity between studies (I2 = 39%, P = 0.07) (Figure 5C). Among the 8 studies (Chen et al., 2022a; Chen et al., 2024; Jinghua et al., 2024; Li et al., 2023; Wu et al., 2016; Yao et al., 2018; Zhang and Liu, 2019; Zhu et al., 2002) reporting implantation rates, ZYP had a significant effect on increasing the implantation rate (RR = 1.19, 95% CI:1.11–1.27, P < 0.00001), and the heterogeneity was low (I2 = 11%, P = 0.35) (Figure 5D). Seventeen RCTs (Chen et al., 2022a; Chen et al., 2024; Gao, 2016; Guo et al., 2024; Jinghua et al., 2024; Li et al., 2023; Lin and Wang, 2024; Liu and Liu, 2020; Wang et al., 2024; Wang et al., 2015; Wu et al., 2016; Yang and Li, 2023; Yang and Yao, 2017; Yao et al., 2018; Zhang and Liu, 2019; Zhou, 2015; Zhu et al., 2002) reported a significant benefit of ZYP in increasing the endometrial thickness on the day of transplantation (MD = 1.06, 95% CI:0.61–1.51, P < 0.00001), but there was large heterogeneity between groups (I2 = 95%, P < 0.00001) (Figure 5E). Eleven studies (Chen et al., 2022a; Gao, 2016; Guo et al., 2024; Jinghua et al., 2024; Li et al., 2023; Wang et al., 2024; Wu et al., 2016; Yang and Yao, 2017; Yao et al., 2018; Zhou, 2015; Zhu et al., 2002) reported the number of oocytes retrieved. The pooled analysis showed that ZYP increased the number of oocytes retrieved (MD = 0.77, 95% CI :0.09–1.45, P = 0.03), but with substantial heterogeneity (I2 = 76%, P < 0.00001) (Figure 5F).

#### 3.3.3 Adverse events

Of the 18 included RCTs, 5 (27.8%) reported adverse event information: 2 studies (Wang et al., 2024; Jinghua et al., 2024) stated that no adverse reactions occurred in either group; 2 studies (Cao et al., 2021; Zhou, 2015) provided specific adverse event counts; and 1 study (Zhu et al., 2002) reported constipation in patient population without providing specific counts. Reported adverse events included nausea/vomiting, joint pain, diarrhea, and rash (Cao et al., 2021); sensation of heat, constipation, acne, and dry mouth (Zhou, 2015); and constipation (Zhu et al., 2002, qualitative only). All events were mild and self-limiting, and no treatment discontinuations were reported. Across the 4 studies with quantifiable data (589 participants; ZYP: n = 244; control: n = 345), adverse event counts were 27 (11.1%) in the ZYP group and 20 (5.8%) in the control group. This numerical difference should be interpreted with caution given the unequal group sizes, small number of events, and lack of standardised adverse event grading. Formal meta-analysis of adverse events was not performed owing to the limited number of reporting studies and heterogeneity in documentation. No serious adverse events, hepatic or renal dysfunction, allergic reactions, or teratogenic effects were reported. The remaining 13 studies (72.2%) did not report adverse event data.

### 3.4 Sensitivity analysis

Sensitivity analyses of each outcome showed robust results for clinical pregnancy rate, biochemical pregnancy rate, miscarriage rate, endometrial thickness, and embryo implantation rate, but unstable results for the number of retrieved oocytes. After excluding the studies by Guo et al. (2024), Yang and Yao (2017), and Li et al. (2023) (Guo et al., 2024; Li et al., 2023; Yang and Yao, 2017) individually. The effect of ZYP on increasing the number of retrieved oocytes became nonsignificant (Supplementary Material 2).

### 3.5 Publication bias

**FIGURE 6:**
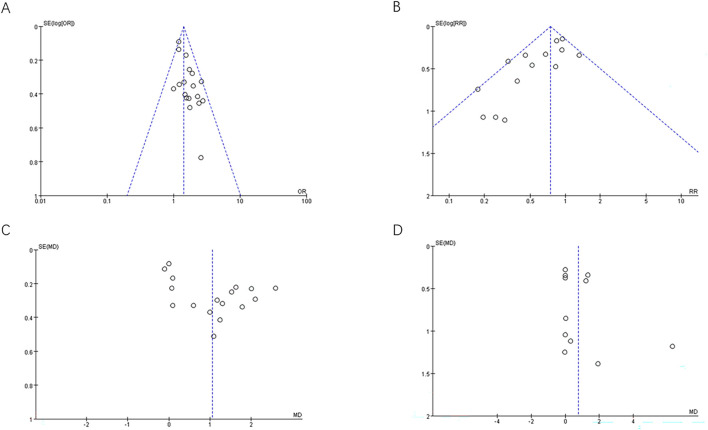
Funnel plots for visual assessment of publication bias. **(A)** Clinical pregnancy rate; **(B)** Miscarriage rate; **(C)** Endometrial thickness on the day of embryo transfer; **(D)** Number of oocytes retrieved.

Egger’s test and funnel plots were constructed for outcomes from more than 10 included studies to assess publication bias. The Egger’s test with a P-value <0.05 (CPR:P = 0.009,MR:P = 0.003,ET:P < 0.001)and asymmetry of the funnel plots of clinical pregnancy rate, miscarriage rate and endometrial thickness suggested publication bias and small study effect, indicating that this review might overestimate the efficacy of ZYP to some extent. This might be because positive results are more likely to be published. No obvious publication bias was observed in the results of the number of retrieved oocytes (Egger’s test with a P = 0.065) (Figure 6).

### 3.6 Quality of evidence

We used GRADE Pro-GDT to evaluate the quality of evidence for each outcome. The results showed that the quality of evidence for each outcome measure was very low. The clinical pregnancy rate, live birth rate, and implantation rate had very low quality of evidence due to certain biases, clinical heterogeneity and risks of publication bias. The quality of evidence for biochemical pregnancy rate was very low due to the few included studies clinical heterogeneity and the low quality of evidence for the risk of publication bias. The quality of evidence for miscarriage rate and endometrial thickness was very low due to bias, publication bias clinical and statistics heterogeneity and inconsistency of results among studies. In addition to the aforementioned reasons for the risk of bias, clinical heterogeneity and inconsistent results among studies, the number of retrieved oocytes was rated as very low due to the wide confidence interval (Table 3).

**TABLE 3:** Certainty of evidence (GRADE).

Outcomes	Quality assessment					No of patients (studies)	Relative (95% CI)	Quality
	Risk of bias	Inconsistency	Indirectness	Imprecision	Publication bias			
Clinical pregnancy rate	serious^1^	serious^2^	No serious indirectness	No serious imprecision	Reporting bias^3^	5,613 (18 studies)	RR 1.22 (1.15–1.3)	⊕ΟΟΟ VERY LOW
Live birth rate	serious^1^	serious^2^	No serious indirectness	No serious imprecision	Reporting bias^3^	4,706 (9 studies)	RR 1.25 (1.15–1.36)	⊕ΟΟΟ VERY LOW
Biochemical pregnancy rate	No serious risk of bias	serious^2^	No serious indirectness	Serious^4^	Reporting bias^3^	3,712 (5 studies)	RR 1.13 (1.05–1.22)	⊕ΟΟΟ VERY LOW
Miscarriage rate	serious^1^	Very serious^2^ ^,^ ^5^	No serious indirectness	No serious imprecision	Reporting bias^3^	2,270 (14 studies)	RR 0.75 (0.63–0.88)	⊕ΟΟΟ VERY LOW
Implantation rate	serious^1^	serious^2^	No serious indirectness	No serious imprecision	Reporting bias^3^	6,547 (8 studies)	RR 1.19 (1.11–1.27)	⊕ΟΟΟ VERY LOW
Endometrium thickness	serious^1^	Very serious^2^ ^,^ ^5^	No serious indirectness	No serious imprecision	Reporting bias^3^	5,566 (17 studies)	—	⊕ΟΟΟ VERY LOW
Number of retrieved oocytes	serious^1^	Very serious^2^ ^,^ ^5^	No serious indirectness	Serious^4^	None	4,004 (11 studies)	—	⊕ΟΟΟ VERY LOW

1 Some studies raised some concerns in randomization and blinding.

2 Existence of clinical heterogeneity.

3 There is publication bias or less negative results.

4 The range of confidence interval is wide or few studies were included.

5 There are some results are inconsistent.

## 4 Discussion

### 4.1 Main findings

Our study indicates that the adjuvant use of ZYP in IVF-ET cycles may lead to a statistically significant improvement in pregnancy outcomes. The 22% relative increase in clinical pregnancy rate (RR = 1.22) and the 25% increase in live birth rate (RR = 1.25) suggest a potentially clinically relevant effect, which could translate to a higher number of successful pregnancies per cycle. The higher RR in the combination subgroup may reflect a potential synergistic effect between ZYP and biomedicine, while the monotherapy subgroup result confirms the independent beneficial effect of ZYP. In addition, ZYP was associated with improved intermediate outcomes, including an increased number of retrieved oocytes and greater endometrial thickness, which may contribute to the observed higher implantation and biochemical pregnancy rates. There were close correlations among implantation rate, biochemical pregnancy rate, and clinical pregnancy rate, as they represented a continuous developmental process from embryo implantation to early clinical pregnancy, and ZYP has a good promoting trend for this continuous development process. Concurrently, a reduction in the miscarriage rate was also noted. These positive effects also significantly increase the chances of successful pregnancy in IVF-ET.

Moreover, few studies reported ZYP’s effects on ectopic pregnancy or preterm birth, and reports on high-quality embryo quality were inconsistent. Thus, we conducted a descriptive analysis of these outcomes. 5 studies (Chen et al., 2022a; Chen et al., 2024; Li et al., 2023; Liu and Liu, 2020; Zhang and Liu, 2019) reported ectopic pregnancy, of which 4 studies (Chen et al., 2024; Li et al., 2023; Liu and Liu, 2020; Zhang and Liu, 2019) found no difference in the incidence of ectopic pregnancy between the ZYP group and the control group, and 1 study (Chen et al., 2022a) found no ectopic pregnancy in the ZYP group and 3 cases in the control group. Among the three studies reporting preterm birth (Cao et al., 2021; Chen et al., 2022a; Li et al., 2023), one study (Cao et al., 2021) demonstrated that ZYP could reduce the incidence of preterm birth, while the other two studies (Chen et al., 2022a; Li et al., 2023) revealed no significant differences compared with the control group. There were 8 RCTs (Cao et al., 2021; Chen et al., 2022a; Gao, 2016; Guo et al., 2024; Li et al., 2023; Wang et al., 2024; Yao et al., 2018; Zhou, 2015) that reported the effect of ZYP on quality embryos, 7 studies (Cao et al., 2021; Gao, 2016; Guo et al., 2024; Li et al., 2023; Wang et al., 2024; Yao et al., 2018; Zhou, 2015) that showed that ZYP increased the rate of quality embryos compared to the control group, but 1 study (Chen et al., 2022a) found no significant difference. In conclusion, despite some promising trends, the current evidence is insufficient to definitively conclude that ZYP can enhance the number of high-quality embryos and reduce the occurrence of ectopic pregnancy and premature birth. Well-designed, large-scale RCTs are urgently needed to clarify these associations.

### 4.2 Mechanism of ZYP

In Traditional Chinese Medicine (TCM) theory, the kidney governs reproduction and stores innate essence. Both oocytes and sperm belong to the category of innate essence, which is derived from essence and blood. The intensive process of controlled ovarian stimulation in IVF treatment, which uses superovulation drugs to harvest multiple mature oocytes in a short period, is believed to consume substantial “kidney essence” (a concept analogous to reproductive potential in the reproductive system). Furthermore, this is often compounded by the fact that patients undergoing IVF treatment may already present with a natural deficiency of “kidney qi” due to advanced age or long-standing infertility. Additionally, the emotional burden of constant worry and excessive mental strain—common in this patient population—can impede the function of the “spleen,” which governs the transformation and transportation of nutrients. Consequently, the comprehensive TCM therapeutic principle of ZYP focuses on “fortifying the spleen and nourishing the kidneys” to restore the physiological balance necessary to support the reproductive system. While these TCM concepts provide a traditional theoretical framework for the clinical application of ZYP, the following discussion focuses on the pharmacological and clinical evidence supporting its effects through the lens of modern biomedical research.

The beneficial effects of ZYP on IVF-ET outcomes may be mediated through several plausible mechanisms. Firstly, ZYP may exert a regulatory effect on endocrine levels. A clinical comparative study demonstrated that ZYP can significantly improve β-HCG, progesterone, and estradiol levels in patients with threatened abortion, thereby enhancing pregnancy success rates (Li et al., 2024). This hormonal regulatory effect is further supported by basic experimental research: an *in vivo* study in ovariectomized rats showed that *Pleuropterus multiflorus (Thunb.)* can regulate sex hormone levels (Zhu et al., 2017), and modern pharmacological studies have confirmed that several “kidney-tonifying” botanical components in ZYP, such as *Eucommia ulmoides* and *Cuscuta chinensis*, possess estrogen-like activities (Zhao et al., 2017). Secondly, ZYP appears to enhance endometrial receptivity by improving uterine blood flow, optimizing endometrial morphology, and increasing endometrial thickness. Consistent with the findings in the existing literature, our meta-analysis further confirmed that ZYP significantly increases endometrial thickness on the day of embryo transfer. Mechanistically, a well-designed *in vivo* study using a mouse model of COH demonstrated that ZYP ameliorates precocious endometrial maturation through upregulation of HOXA10, a key gene involved in embryo implantation (Gao et al., 2015). Further clinical studies have reported that ZYP promotes the formation of a “type A″ endometrial pattern and improves uterine hemodynamics by regulating vascular endothelial factors (e.g., VEGF and VEGFR-2) as well as reducing the RI and PI of the endometrium (Chu et al., 2018; Jiang et al., 2022; Liu et al., 2016; Wang et al., 2015; Zhang and Liu, 2019). Thirdly, ZYP is likely to modulate the immune microenvironment to maintain maternal-fetal tolerance, which is essential for the maintenance of normal pregnancy. A clinical observational study reported that ZYP can help restore the balance of Th17/Treg cells in patients with recurrent pregnancy loss (Zou and Zhang, 2019), and this balance is a crucial prerequisite for sustaining a stable pregnancy. The broader biological plausibility of ZYP’s immunomodulatory role has been further discussed by Maharajan et al. (2021).

Nonetheless, certain limitations of the current mechanistic evidence should be acknowledged. The proposed mechanisms are predominantly derived from preclinical studies (animal models and *in vitro* experiments) and small-scale clinical observations. To date, no large-scale, mechanistically focused randomized controlled trial has been conducted to definitively validate these pathways specifically in the IVF-ET population. Furthermore, the quality and generalizability of some Chinese-language references, while methodologically sound, may be limited by the scope of their study designs. Future studies incorporating translational designs—integrating pharmacokinetic profiling, biomarker analysis, and mechanistic endpoints within well-powered clinical trials—are warranted to establish causal relationships between ZYP administration and the proposed biological mechanisms.

### 4.3 Safety

Safety reporting in the included trials was sparse: only 5 of 18 studies (27.8%) documented adverse events, and only 4 provided quantifiable data. All reported events were mild (gastrointestinal, dermatological, or musculoskeletal symptoms), with no serious adverse events in any trial. The adverse event incidence was numerically higher in the ZYP group (11.1% vs. 5.8%); however, unequal group sizes, the small number of events, absence of standardised grading, and substantial reporting bias (13/18 studies silent on safety) preclude definitive interpretation. These observations are consistent with Maharajan et al. (2021), who reported general tolerability of ZYP in preclinical toxicological studies (Maharajan et al., 2021).

The safety evidence gap is of particular concern for the IVF-ET population, in whom supraphysiological hormone exposure increases hepatic and renal metabolic demands, and developing embryos are vulnerable to pharmacological perturbation. Key unresolved questions include: (1) interactions between ZYP and ovulation-inducing agents (gonadotropins, GnRH analogues, progesterone); (2) hepatic and renal effects during concurrent hormonal stimulation; and (3) long-term offspring safety. Future trials should incorporate safety as a primary outcome, employ standardised grading (e.g., CTCAE), monitor hepatic and renal function, and extend follow-up to neonatal outcomes.

### 4.4 Heterogeneity analysis

1. The high heterogeneity observed for endometrial thickness and number of retrieved oocytes may stem from several factors. First, differences in patients’ baseline age likely contribute, as ovarian reserve declines with advancing age, potentially leading to divergent responses to ZYP. Second, variability in ovarian stimulation protocols—such as the long agonist protocol, GnRH antagonist protocol, and mild stimulation—creates distinct hormonal environments, which could either enhance or mask the effects of ZYP. Third, the timing of ZYP administration varies across studies: early-phase administration primarily influences follicular development, whereas late-phase administration is more likely to affect endometrial receptivity. Pooling studies with different administration timings may dilute or disperse the effect estimates for these outcomes. Furthermore, the measurement methods and operational definitions of these outcomes varied across trials. Collectively, these factors represent the major sources of the observed heterogeneity. Therefore, the current pooled results for endometrial thickness and number of retrieved oocytes should be interpreted as approximate trends under conditions of substantial heterogeneity, rather than as precise effect estimates; these findings should be regarded as exploratory and interpreted with considerable caution. Future studies should adopt standardized intervention protocols, targeting specific age groups, employing uniform ovarian stimulation regimens, and clearly defining the timing of ZYP administration, to help clarify the true effect of ZYP on these secondary outcome measures. In addition, only five studies reported the biochemical pregnancy rate, and thus the possibility of underestimating heterogeneity for this outcome cannot be ruled out.
2. The methodological quality of the included studies is heterogeneous. Some trials provided insufficient details regarding aspects such as random sequence generation, allocation concealment, and blinding implementation. This may lead to publication bias and lowers the evidence level. It should be noted that the methodological limitations of these studies—including insufficient reporting of random sequence generation, allocation concealment, and blinding—could have influenced the treatment effect evaluation, with a potential risk of overestimating the effect size. These limitations, combined with evidence of publication bias, mean that the true efficacy of ZYP may be substantially lower than suggested by the pooled estimates. Future randomized controlled trials should strictly adhere to the CONSORT (Hopewell et al., 2025) statement for reporting to enhance research transparency and accuracy.
3. The heterogeneity of interventions is increased by variations in the initiation timing and duration of oral ZYP administration across different randomized controlled trials. This is particularly significant given the complex physiological processes of IVF-ET. When administered during the follicular phase, ZYP primarily influences follicular recruitment and growth, thereby increasing the number of oocytes retrieved. In contrast, intervention during the peri-implantation or luteal phase mainly aims to enhance endometrial receptivity, improving implantation and clinical pregnancy rates. The effects differ substantially if the medication spans the follicular phase, the luteal phase, or the entire treatment cycle. Additionally, the duration of administration is closely associated with the cumulative drug effect. Among the included studies, treatment courses varied widely, ranging from 3 weeks to 3 months, indicating that interventions were not uniformly defined. This suggests that the analyzed outcomes may reflect an average effect of ZYP use at “any time point” rather than the optimal timing, introducing significant heterogeneity and reducing the certainty and specificity of the pooled results. Future studies should focus on standardizing ZYP intervention protocols by specifying clear initiation timepoints and fixed treatment durations to identify the optimal therapeutic window and regimen.
4. The study population included patients with PCOS, DOR, and RIF, undergoing both fresh and frozen cycles, demonstrating significant clinical heterogeneity. Despite low statistical heterogeneity (I^2^ ≤ 50%) in most outcome measures, the diverse etiologies and pathophysiological mechanisms necessitate careful consideration. For example, in PCOS patients, ZYP may primarily regulate hormone levels and follicular development, whereas in RIF patients, its action might be more focused on modulating endometrial receptivity and the microenvironment. For DOR patients, its main effects likely involve hormonal regulation, improvement of oocyte quality, and enhancement of ovarian response.

From the perspective of Traditional Chinese Medicine’s holistic approach, ZYP might achieve common downstream effects, such as regulating the HPO axis, improving follicular quality, and enhancing endometrial receptivity, across different etiologies. However, the specific pathways, magnitude of effects, and clinical relevance may differ substantially. Due to the limited number of available studies and the heterogeneous reporting of etiology-specific data, it was not feasible to conduct meaningful subgroup analyses stratified by infertility etiology. Notably, statistical homogeneity does not equate to clinical homogeneity. The pooled estimates may mask clinically meaningful differential effects, potentially overestimating efficacy in certain subgroups while underestimating it in others. The current pooled results reflect an average treatment effect of ZYP across a broad, mixed population rather than a precise evaluation within specific subgroups, which inherently limits the generalizability of the conclusions to any single patient population. Future research should target specific patient populations and standardized cycle protocols to more accurately determine the optimal indications for ZYP.

### 4.5 GRADE

According to the GRADE approach, the certainty of evidence for ZYP’s efficacy and safety in IVF-ET adjunctive therapy was very low across all outcomes, substantially limiting the clinical applicability of our pooled results. This downgrading was driven by two key factors: high clinical and methodological heterogeneity (variable infertility etiologies, non-standardized ZYP administration, and divergent stimulation protocols), and publication bias (identified via Egger’s test and funnel plot asymmetry for key outcomes such as clinical pregnancy rate) from predominantly positive, single-center China-based trials. Although our meta-analysis showed statistically significant associations between ZYP and improved IVF-ET outcomes, these findings must be interpreted with considerable caution and cannot support routine clinical use, as the very low evidence certainty suggests that the true effect of ZYP may differ substantially from our pooled estimates. Future research should focus on large-scale, multicenter, double-blind RCTs with standardized protocols and mandatory reporting of all trial results regardless of outcome to generate reliable higher-certainty evidence.

### 4.6 Strengths and limitations

This study represents the first systematic review and meta-analysis of randomized controlled trials to evaluate ZYP’s impact on IVF-ET pregnancy outcomes. Our finding that ZYP significantly improves the clinical pregnancy rate (RR = 1.22, 95% CI: 1.15–1.30) quantitatively supports and aligns with the conclusions of a previous narrative review by Maharajan et al. (2021), which also suggested ZYP’s efficacy in IVF (Maharajan et al., 2021). Furthermore, we strictly included randomized controlled trials in accordance with the PICOS eligibility criteria, thereby enhancing the reliability of the meta-analysis.

However, this study has several limitations that require cautious interpretation of its findings. First, methodological flaws in the included trials—insufficient reporting of random sequence generation, allocation concealment, and blinding, together with significant publication bias and small-study effects for key outcomes—likely led to overestimation of ZYP’s beneficial effects on IVF-ET outcomes. According to the GRADE approach, the certainty of evidence was very low across all outcomes, substantially limiting the clinical applicability of our pooled results. Second, inconsistent ZYP initiation timing and duration across RCTs increased intervention heterogeneity. Third, limited stratification data precluded important subgroup analyses: most studies did not specify whether fresh or frozen embryos were transferred or whether participants had a history of recurrent implantation failure (RIF), making it impossible to explore ZYP’s differential efficacy by these factors. Fourth, all trials were conducted in China, restricting the generalizability of pooled effect sizes to other populations given global variations in IVF protocols and practices; our findings, though encouraging and promising, have not yet changed clinical practice and should not be extrapolated to non-Chinese populations without validation.

Additionally, inconsistent miscarriage definitions across trials—with some reporting only early pregnancy loss and others combining early and late loss without distinction—introduced classification bias and reduced the reliability of pooled estimates, although definitions for other primary outcomes (e.g., clinical pregnancy rate, implantation rate) were generally consistent.

Finally, safety evidence was severely inadequate: only 5 of 18 studies reported adverse events, with 4 providing quantitative data and 1 reporting qualitative information only (Zhu et al., 2002). The numerically higher adverse event rate in the ZYP group (11.1% vs. 5.8% in controls), derived from 589 participants with unequal group allocation, underscores the critical need for systematic safety monitoring. No study assessed hepatic or renal function, potential drug interactions between ZYP and ovulation-inducing agents, or long-term offspring outcomes—critical gaps given the unique vulnerabilities of the IVF-ET population.

Consequently, given the above limitations, the conclusions of this study should be regarded as encouraging and promising, but have not yet been sufficient to change clinical practice. At the same time, it should be carefully extrapolated to other ethnic groups. Future research should involve international, multi-center trials to verify the applicability and effectiveness of ZYP in diverse global IVF populations.

## 5 Conclusion

In conclusion, this meta-analysis demonstrates that ZYP, as an adjunctive treatment, is associated with a positive trend in improving pregnancy outcomes among women undergoing IVF (e.g., clinical pregnancy rate with a RR of 1.22). However, given the low certainty of evidence and the risk of bias in the included studies, this finding cannot yet support changes in clinical practice and should be regarded as exploratory. Future research should prioritize large-scale, methodologically rigorous, double-blind, placebo-controlled randomized trials to clearly establish the efficacy and safety of ZYP across different IVF-ET populations.

## Data Availability

The original contributions presented in the study are included in the article/Supplementary Material, further inquiries can be directed to the corresponding authors.
