# Supplementary material for: Effects of the commercial Chinese polyherbal preparation Zishen Yutai Pill on the pregnancy outcomes in women undergoing in vitro fertilization-embryo transfer: a systematic review and meta-analysis of randomized controlled trials
Source: Front Pharmacol. 2026 Apr 23;17:1770841. doi: 10.3389/fphar.2026.1770841 (PMC13149295; doi:10.3389/fphar.2026.1770841)
Supplement: Supplementary file 1 [file Supplementaryfile1.docx]

***Search strategy***

**Pubmed**

((((((((((embryo transfer) OR (embryo transfer[MeSH Terms])) OR (embryo transfer[Title/Abstract])) OR (Embryo Transfers[MeSH Terms])) OR (Transfer, Embryo[MeSH Terms])) OR (Transfers, Embryo[MeSH Terms])) OR (Blastocyst Transfer[MeSH Terms])) OR (Tubal Embryo Transfer[MeSH Terms])) OR (Tubal Embryo Stage Transfer[MeSH Terms])) OR ((((((((((((((((Fertilization in Vitro) OR (Fertilization in Vitro[MeSH Terms])) OR (Fertilization in Vitro[Title/Abstract])) OR (In Vitro Fertilization[MeSH Terms])) OR (In Vitro Fertilizations[MeSH Terms])) OR (Test-Tube Fertilization[MeSH Terms])) OR (Fertilization, Test-Tube[MeSH Terms])) OR (Fertilizations, Test-Tube[MeSH Terms])) OR (Test Tube Fertilization[MeSH Terms])) OR (Test-Tube Fertilizations[MeSH Terms])) OR (Fertilizations in Vitro[MeSH Terms])) OR (Test-Tube Babies[MeSH Terms])) OR (Babies, Test-Tube[MeSH Terms])) OR (Baby, Test-Tube[MeSH Terms])) OR (Test Tube Babies[MeSH Terms])) OR (Test-Tube Baby[MeSH Terms]))) AND (((Zishen Yutai ) OR (Zishen Yutai [MeSH Terms])) OR (Zishen Yutai [Title/Abstract]))

**Web of Science**

#1 ((TS=(embryo transfer OR Embryo OR Transfers OR Embryo OR Transfers OR Tubal Embryo Transfer OR Tubal Embryo Transfer)) OR TI=(embryo transfer OR Embryo OR Transfers OR Embryo OR Transfers OR Tubal Embryo Transfer OR Tubal Embryo Transfer)) OR AB=(embryo transfer OR Embryo OR Transfers OR Embryo OR Transfers OR Tubal Embryo Transfer OR Tubal Embryo Transfer)

#2 (TS=(Fertilization in Vitro OR In Vitro Fertilization OR In Vitro Fertilizations OR Test-Tube Fertilization OR Fertilization, Test-Tube OR Fertilizations OR Test Tube Fertilization OR Test-Tube Babies OR Babies OR Baby)) OR TI=(Fertilization in Vitro OR In Vitro Fertilization OR In Vitro Fertilizations OR Test-Tube Fertilization OR Fertilization, Test-Tube OR Fertilizations OR Test Tube Fertilization OR Test-Tube Babies OR Babies OR Baby) OR AB=( Fertilization in Vitro OR In Vitro Fertilization OR In Vitro Fertilizations OR Test-Tube Fertilization OR Fertilization, Test-Tube OR Fertilizations OR Test Tube Fertilization OR Test-Tube Babies OR Babies OR Baby)

#3 (TS=(Zishen Yutai OR Zishen Yutai Pill OR Zishen Yutai Pills OR zishen yutai ) )OR (TI=( Zishen Yutai OR Zishen Yutai Pill OR Zishen Yutai Pills OR zishen yutai ) )OR (AB=( Zishen Yutai OR Zishen Yutai Pill OR Zishen Yutai Pills OR zishen yutai ))

((#1) OR #2) AND #3

**The Cochrane Library**

ID Search Hits

#1 MeSH descriptor: [Embryo Transfer] explode all trees 1579

#2 MeSH descriptor: [Fertilization in Vitro] explode all trees 3015

#3 Zishen Yutai 27

#4 embryo transfer 5980

#5 Zishen Yutai pill 25

#6 #1 OR #2 OR #4 7313

#7 #3 OR #5 27

#8 #6 AND #7 14

**Embase**

('embryo transfer'/exp OR 'embryo transplantation' OR 'transfer, embryo' OR 'embryo transfer' OR 'in vitro fertilization'/exp OR 'extracorporeal fertilization' OR 'fertilization in vitro' OR 'in vitro fertilisation' OR 'ivf (in vitro fertilization)' OR 'testtube baby' OR 'in vitro fertilization') AND 'zishen yutai'/exp

**CNKI：(https://www.cnki.net/old/)**

((SU='体外受精' OR SU='体外受精-胚胎移植' OR SU='体外受精-胚胎移植术' OR SU='体外受精胚胎移植') OR (SU='试管婴儿' OR SU='试管婴儿技术') OR (SU='胚胎移植' OR SU='胚胎移植技术' OR SU='胚胎移植术' OR SU='胚胎移植(et)')) AND (SU='滋肾育胎' OR SU='滋肾育胎丸' OR SU='滋肾育胎方')

**Wanfang：(https://s.wanfangdata.com.cn/nav-page?a=second)**

（全部:(体外受精\-胚胎移植) or 全部:(体外受精) or 全部:(胚胎移植) ）and 全部:(滋肾育胎)

**VIP：(https://qikan.cqvip.com/)**

((M=体外受精-胚胎移植 OR M=体外受精 OR M=胚胎移植) OR (U=体外受精-胚胎移植 OR U=体外受精 OR U=胚胎移植 )) AND ((M=滋肾育胎) OR (U=滋肾育胎))

**CBM：(https://www.sinomed.ac.cn/index.jsp)**

((("体外受精-胚胎移植"[全部字段:智能] OR "体外受精"[全部字段:智能] OR "胚胎移植"[全部字段:智能])) AND (("滋肾育胎丸"[全部字段:智能] OR "滋肾育胎"[全部字段:智能])))
