# Supplementary material for: Effects of the commercial Chinese polyherbal preparation Zishen Yutai Pill on the pregnancy outcomes in women undergoing in vitro fertilization-embryo transfer: a systematic review and meta-analysis of randomized controlled trials
Source: Front Pharmacol. 2026 Apr 23;17:1770841. doi: 10.3389/fphar.2026.1770841 (PMC13149295; doi:10.3389/fphar.2026.1770841)
Supplement: Supplementary file 2 [file Supplementaryfile2.docx]

***Sensitivity analysis of number of oocytes retrieved***

| **Number of oocytes retrieved** | **RR (95% CI)** | **I^2^** | **P** |
| --- | --- | --- | --- |
| Original analysis | RR=0.77,95% CI [0.09,1.45] | 76% | 0.03 |
| Exclude Guo XL 2024 | RR=0.45,95% CI [-0.01,0.91] | 47% | 0.05 |
| Exclude Yang XJ 2017 | RR=0.72,95% CI [-0.03,1.48] | 76% | 0.06 |
| Exclude Li XF 2023 | RR=0.69,95% CI [-0.05,1.43] | 74% | 0.07 |
